# Cool-Climate Red Wines—Chemical Composition and Comparison of Two Protocols for ^1^H-NMR Analysis

**DOI:** 10.3390/molecules23010160

**Published:** 2018-01-13

**Authors:** Violetta Aru, Klavs Martin Sørensen, Bekzod Khakimov, Torben Bo Toldam-Andersen, Søren Balling Engelsen

**Affiliations:** 1 Department of Food Science, University of Copenhagen, Rolighedsvej 26, DK-1958 Frederiksberg, Denmark; violetta@food.ku.dk (V.A.); kms@food.ku.dk (K.M.S.); bzo@food.ku.dk (B.K.); 2 Department of Plant and Environmental Sciences, University of Copenhagen, Højbakkegård Alle 13, DK-2630 Taastrup, Denmark; tbta@plen.ku.dk

**Keywords:** ^1^H-NMR, FT-IR, cool-climate wines, Danish wines, LalVigne Mature, Pometum, wine as-is, dried wines, PCA

## Abstract

This study investigates the metabolome of 26 experimental cool-climate wines made from 22 grape varieties using two different protocols for wine analysis by proton nuclear magnetic resonance (^1^H-NMR) spectroscopy. The wine samples were analyzed as-is (wet) and as dried samples. The NMR datasets were preprocessed by alignment and mean centering. No normalization or scaling was performed. The “wet” method preserved the inherent properties of the samples and provided a fast and effective overview of the molecular composition of the wines. The “dried” method yielded a slightly better sensitivity towards a broader range of the compounds present in wines. A total of 27 metabolites including amino acids, organic acids, sugars, and alkaloids were identified in the ^1^H-NMR spectra of the wine samples. Principal component analysis was performed on both NMR datasets evidencing well-defined molecular fingerprints for ‘Baco Noir’, ‘Bolero’, ‘Cabernet Cantor’, ‘Cabernet Cortis’, ‘Don Muscat’, ‘Eszter’, ‘Golubok’, ‘New York Muscat’, ‘Regent’, ‘Rondo’, ‘Triomphe d’Alsace’, ‘Précose Noir’, and ‘Vinoslivy’ wines. Amongst the identified metabolites, lactic acid, succinic acid, acetic acid, gallic acid, glycerol, and methanol were found to drive sample groupings. The ^1^H-NMR data was compared to the absolute concentration values obtained from a reference Fourier transform infrared method, evidencing a high correlation.

## 1. Introduction

Grape wine is a beverage made from the fermented juice of grapes whose chemical composition consists of inorganic and organic molecules of diverse nature including water, alcohols, proteins, amino acids, carbohydrates, and polyphenols [1]. Recent research has shown that a regular and moderate consumption of red wine is associated with health benefits [2,3,4]. In particular, many research and epidemiological studies have brought evidence that the intake of polyphenols as grape juice and red wine is associated with a reduced risk of cardio vascular diseases [5,6,7,8], increased lifespan due to resveratrol, a stilbenoid found in red grapes, which is proposed to prevent the age-related decline in the cardiovascular function [9,10], and a number of other beneficial health effects [11].

Besides defining the quality and character of the beverage, the chemical composition of wines reflects the history of the wine making process, including the yeast strain, the grape variety, the fermentation process, and storage, as well as the geographic origin [12,13,14,15,16,17]. According to the statistics reported by the International Organization of the Vine and Wine (OIV), the leading wine producers remain the Mediterranean countries of Europe where the fertile soils and the mitigate climate promote optimal fruit maturity. However, despite unfavorable factors such as the shorter growing season, lower sunshine duration, higher humidity and risk of frost, Northern European countries have recently emerged as new wine producing countries. During the last decades, the development and intensive breeding of new disease-resistant grape varieties has brought the limit for vine growing to lower latitudes [18]. In particular, Denmark has been counted as one of the European vine-growing countries since 2000 [18]. Many attempts have been made in order to introduce and improve viticulture in Denmark, from the development of a method for identifying potential cool-climate vine growing sites [19] over the finding of suitable cultivars, the best vinification methods and wine styles, to the identification of the best yeast strains for the production of high-quality cool-climate wines [20].

Analytical chemistry is increasingly playing an important role in the wine industry, as the chemical analysis of wine is an essential tool for ensuring product quality as well as for elucidating many aspects related to grape and wine production [21,22]. Nuclear magnetic resonance (NMR) spectroscopy is a powerful analytical platform that is widely applied as a high-throughput method for the analysis of biological samples, since it requires minimal sample preparation [23]. NMR spectroscopy is an unbiased and effective screening tool able to capture a snapshot of primary metabolites (i.e., sugars, organic acids, amino acids) and secondary metabolites (i.e., flavonoids, anthocyanins, and other pigments) in the sample under investigation. It has successfully been used for the overall chemical characterization of wines and several grape-derived products [24], as well as in wine metabolomics investigations with the purpose to study the effect of vintage [16], berry shading [15], geographical origin [12], and the alcoholic and malolactic fermentation processes [25]. By using NMR spectroscopy, many different molecules have been found in wines including amino acids (i.e., leucine, isoleucine, valine, threonine, alanine, arginine, glutamine, proline, and tyrosine), organic acids (i.e., succinic acid, acetic acid, malic acid, tartaric acid, and citric acid), sugars (α- and β-glucose, fructose, and arabinose), 2,3-butanediol, glycerol, 2-phenylethanol, trigonelline, and phenylpropanoids (cis/trans-caftaric acid, cis/trans-caffeoyl malate, and cis/trans-coutaric acid) [26,27].

In general, no sample pre-treatment is required prior to untargeted NMR analysis of wine [27]. However, pH adjustment and/or buffer addition are usually performed to minimize the signal shifts in the NMR spectra due to pH fluctuations amongst different wine samples [28]. Lyophilization and multiple solvent suppression can be used to remove the strong and dominating signals of water and ethanol [29,30]. When performing targeted NMR analysis, for example of phenolic compounds, metabolites extraction must be carried out [31,32].

This work aimed to develop an efficient, sensitive, and reproducible method for an optimal ^1^H-NMR analysis of wines by comparing two different sample preparation protocols. For this purpose, 26 experimental cool-climate wines, made from 22 different grape varieties harvested in Denmark during vintage 2016, were measured using both methods. The efficiency of the two protocols was assessed by comparison of the ^1^H-NMR method with a reference Fourier transform infrared (FT-IR) method.

## 2. Results

2.1. ^1^H-NMR Spectroscopy

**Figure 1:**
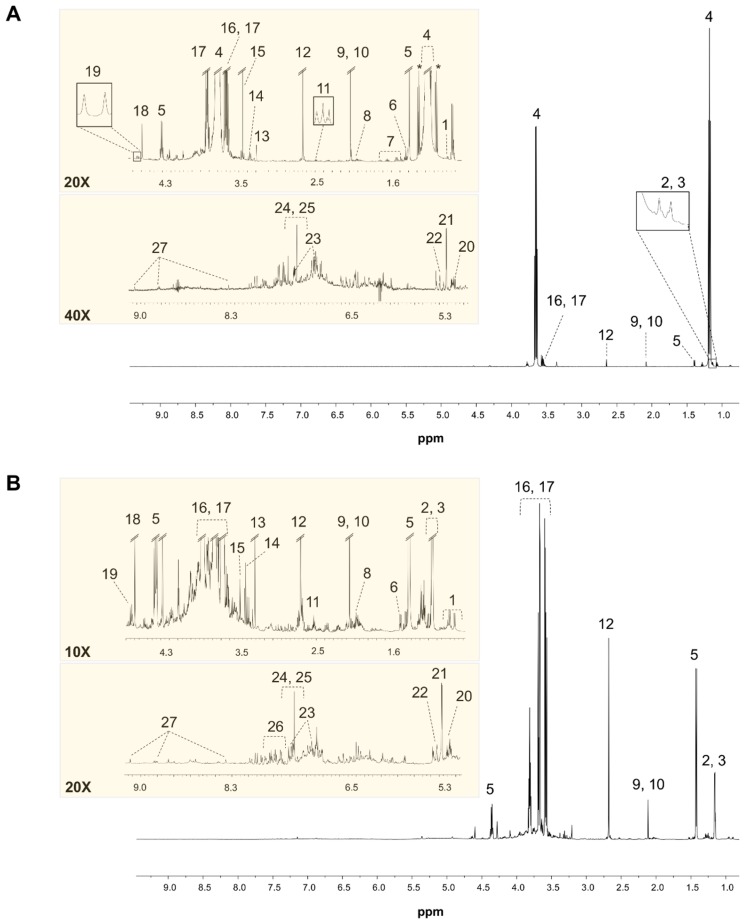
Representative ^1^H-NMR spectra of wet (**A**) and dried wine (**B**) samples. The major resonances have been assigned: 1. Valine; 2. 2,3-butanediol; 3. Isopropanol; 4. Ethanol; 5. Lactic acid; 6. Alanine; 7. Arginine; 8. Proline; 9. Acetic acid; 10. Methionine; 11. γ-aminobutyric acid; 12. Succinic acid; 13. Choline; 14. Myoinositol; 15. Methanol; 16. Fructose; 17. Glycerol; 18. Tartaric acid; 19. β-glucose; 20. α-glucose; 21. Arabinose; 22. Cis-caftaric acid; 23. Tyrosine; 24. Gallic acid; 25. 2-phenyethanol; 26. Phenylalanine; 27. Trigonelline.

The representative ^1^H-NMR spectra of wine as-is (wet) and dried wine samples are shown in Figure 1A,B, respectively. The primary difference between the spectra of the two sample types is the presence of the strong ^1^H resonances of ethanol at 1.18 ppm and 3.65 ppm, and methanol at 3.36 ppm, in the ^1^H-NMR spectra of the wet samples. In the dried samples, where the signals of ethanol and methanol are no longer present, the strongest signals stem from glycerol (multiplets at 3.57, 3.68, and 3.81 ppm). In addition, several organic acids are present in the aliphatic region of the ^1^H-NMR spectra of both samples types, with lactic acid, succinic acid, and acetic acid being the predominant ones. The amino acid pool in the wine samples consists of both polar and non-polar molecules such as proline, alanine, and arginine. Amongst the carbohydrates, α- and β-glucose are identified in the wine samples of both types. The aromatic region of the ^1^H-NMR spectra contains the signals from different molecular classes such as amino acids (i.e., tyrosine), higher alcohols (i.e., 2-phenylethanol), alkaloids (i.e., trigonelline), and phenolic compounds (i.e., gallic acid). Table 1 shows the multiplicity and chemical shifts of the assigned metabolites. In total, 27 compounds were identified in the wine samples.

*Protocol quality control.* The efficiency of the applied sample preparation methods was assessed in terms of number of compounds detected in the average ^1^H-NMR spectra of wet and dried wine samples, as well as through the obtained reproducibility. Even though sample up-concentration increased the signal-to-noise in the dried wines spectra, the drying process also led to the loss/decrease of several semi-volatile and volatile metabolites. In particular, the amount of the unknown signal at 0.89 ppm and methanol (**15**) has drastically decreased in the dried wines. As expected, the ratio of acetic acid (**9**) to lactic acid (**5**) in dried wines is reduced to half-level of that in the wet wine samples, while the lactic acid to succinic acid (**12**) and gallic acid (**24**) to arabinose (**21**) ratios remain constant. The signal intensity of the unknown metabolite at 7.30 ppm (singlet) has decreased by a factor of two in the dried samples. The drying process has affected the aromatic region between 6.50 ppm and 7.50 ppm, where several spin systems have disappeared. The relative amount of the least abundant (detectable) metabolite in wine, trigonelline (**27**), was constant in both wet and dried wine samples. After the vacuum drying process, an unknown multiplet emerged at 1.18 ppm where the ethanol signal dominated in the wet wine samples.

### 2.2. Multivariate Analysis

**Figure 2:**
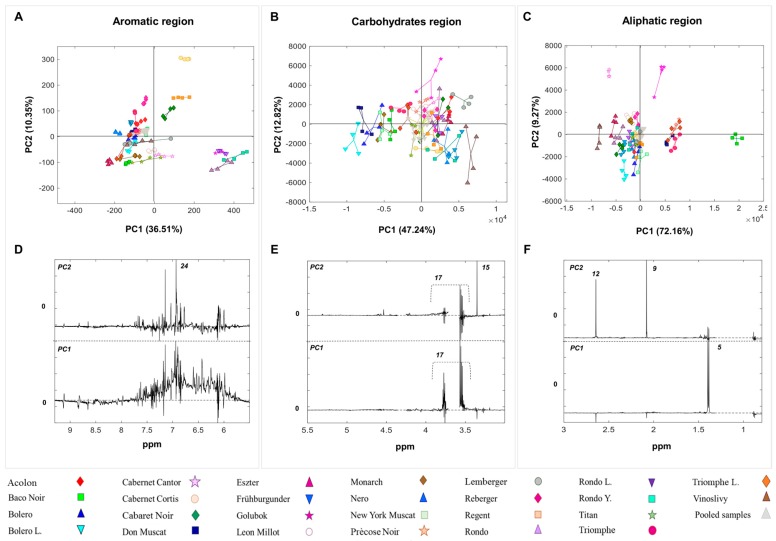
Scores and loadings plots of the PCA on the aliphatic (0.80–3 ppm) (**C**–**F**); carbohydrates (3.01–5.50 ppm) (**B**–**E**); and aromatic (5.51–9.30) (**A**–**D**) regions of the ^1^H-NMR spectra from wet wines. The explained variance, for each principal component, is reported in parenthesis.

**Figure 3:**
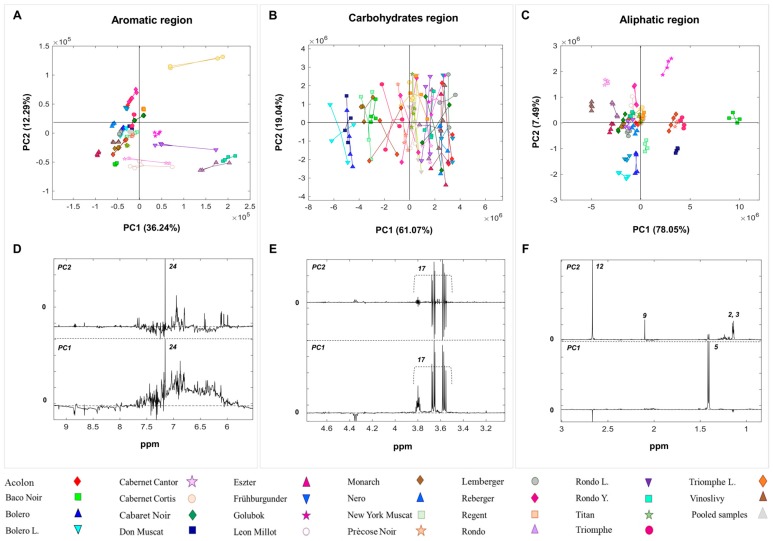
Scores and loadings plots of the PCA on the aliphatic (0.80–3 ppm) (**C**–**F**); carbohydrates (3.01–5.50 ppm) (**B**–**E**); and aromatic (5.51–9.30) (**A**–**D**) regions of the ^1^H-NMR spectra from the dried wines. The explained variance for each principal component is reported in parenthesis.

PCA was performed on the wet and dried wine datasets (Figure 2 and Figure 3). The analysis was carried out separately on three different areas of the ^1^H-NMR spectra labelled the aliphatic region (0.80–3.00 ppm), the carbohydrates region (3.01–5.50 ppm), and the aromatic region (5.51–9.30 ppm) [33]. 

*Aliphatic region.* Figure 2C–F and Figure 3C–F display the scores plots and correspondent loadings plots of the PCA performed on the wet and dried wine datasets, respectively. Overall, the explained systematic variance by the first two PCs is around 81% in the wet wine dataset and 85% in the dried wine dataset. A clear differentiation amongst the wine samples made from different grape varieties can be observed in both scores plots (Figure 2C and Figure 3C). The scores distribution along PC1 is driven by the same metabolites (Figure 2F and Figure 3F), namely lactic acid, and succinic acid in both wine datasets. In particular, ‘Baco Noir’ samples are characterised by high levels of lactic acid, while ‘Vinoslivy’ samples are rich in succinic acid. Interesting, ‘Précose Noir’ and ‘Triomphe d’Alsace’ samples cluster together in the score plots of both PCA models. Samples distribution along PC2 is mainly driven by acetic acid and succinic acid in both datasets. These metabolites are particularly abundant in ‘Cabernet Cantor’ and ‘Golubok’ samples. Except for ‘Golubok’ (0.79 g/L), the levels of acetic acid, as measured by the WineScan, were in the normal range for red wines (0.2–0.5 g/L) (data not shown). The metabolite 2,3-butanediol is found to play a key role in the scores distribution of the dried wine dataset.

It is worth mentioning that, due to the removal of the ethanol signals in the wet wine data, the PCA models developed using the aliphatic regions were built using a different number of variables in the two wine datasets. Nevertheless, the results of the PCA were similar.

*Carbohydrates region.*Figure 2B–E and Figure 3B–E show the scores and loadings plots of PCA applied to the carbohydrates regions of the ^1^H-NMR spectra from wet and dried wine samples, respectively. A total of 60.06% and 80.11% of the systematic variation is explained by the first two PCs, in the wet and dried wine datasets, respectively. This region proved to be the least informative in terms of sample clustering according to different grape varieties in both datasets. However, a consistent trend is observed in both wet and dried wines where ‘Bolero’, ‘Bolero L.’, and ‘Don Muscat’ samples show the lowest glycerol content. The ‘Golubok’ samples have the highest content of methanol (Figure 2B–E).

*Aromatic region.* Figure 2A–D and Figure 3A–D display the PC1 versus PC2 scores and loadings of the PCA models performed on the aromatic region of the ^1^H-NMR spectra of the wet and dried wine samples, respectively. PC1 and PC2 explain a cumulative variance of 46.86% and 48.53% in the wet and dried wine datasets, respectively. A remarkable grouping of wine types is observed in the scores plots, indicating a well-defined molecular fingerprint in each of the wine samples. All ‘Rondo’ samples, namely ‘Rondo’, ‘Rondo Y.’, and ‘Rondo L.’, cluster together in the score plot obtained from the PCA on the wet dataset. Along with the ‘Golubok’ samples, all ‘Rondo’ samples are characterised by a high concentration of phenolic compounds (Figure 2A–D).

The pooled wine samples, which represent the chemical average of all samples, are clustered in the center of the scores plots and validate the high reproducibility of the sample protocol.

### 2.3. Quality Control of ^1^H-NMR Data

**Figure 4:**
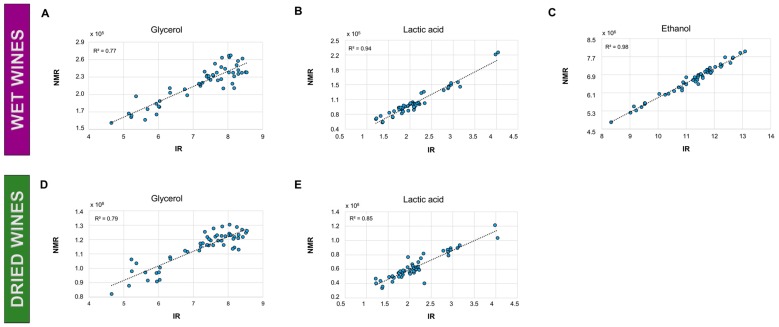
Correlations of NMR (intensities/integrals) to IR calibrated concentrations (WineScan). NMR-IR correlations for glycerol (**A**); lactic acid (**B**); and ethanol (**C**) as measured in wet wine samples; NMR-IR correlations for glycerol (**D**) and lactic acid (**E**) as measured in the dried wine samples.

Lactic acid, glycerol, and ethanol were chosen as target molecules for the quality control of the wet wine samples. The regression models in Figure 4B,C show correlations between the IR and ^1^H-NMR data for lactic acid (*R*^2^ = 0.94 ) and ethanol (*R*^2^ = 0.98), as measured in the wet wine samples. A weaker correlation is observed in the case of glycerol: *R*^2^ = 0.77 (Figure 4A). Concerning the dried wine samples, lactic acid and glycerol were chosen as target molecules. Correlations between ^1^H-NMR and IR data were 0.79 and 0.85 for glycerol and lactic acid, respectively (Figure 4D,E).

**Figure 5:**
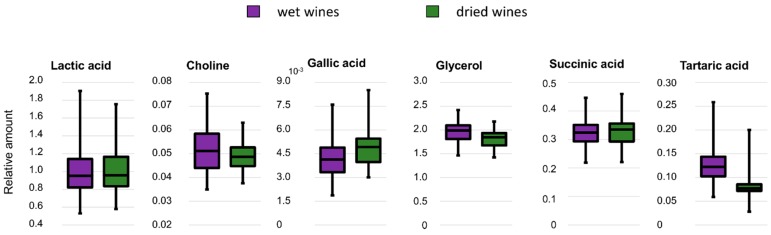
Box and whiskers plots showing the variability distribution of lactic acid, choline, gallic acid, glycerol, succinic acid, and tartaric acid in the wet and dried wine sample sets. Purple boxes are the wet wine samples and the green boxes are the dried wine samples. The wet and the dry datasets have been scaled to the same average intensity of lactic acid.

In order to assess the variability distribution in the wine samples, the relative amount of 6 representative metabolites, namely lactic acid, succinic acid, choline, glycerol, tartaric acid, and gallic acid, were calculated in the ^1^H-NMR spectra of wet and dried wines. As shown in Figure 5, the two sample preparation methods yield comparable distribution, albeit differences can be observed for the individual metabolites. In particular, the recovery in dried samples was less efficient for tartaric acid.

## 3. Discussion

Two different protocols for wine sample preparation prior to ^1^H-NMR analysis were compared using a total of 26 experimental wine samples. In the first method, wine was measured directly after addition of phosphate buffer (wet wines). The advantage of this approach is its simplicity (no drying and no titration), but the high concentration of ethanol limited the dynamic range in the NMR spectrometer (receiver gain), resulting in low signal-to-noise ratio of minor compounds. Despite being dominated by the presence of the two ethanol signals, the obtained ^1^H-NMR spectra of the wet samples provided an overview of the chemical composition of the wines. In the second method, the wine samples were dried and resolubilized in phosphate buffer. The advantage of this method is the removal of the intense ethanol signals, a possible up-concentration, and, possibly, a much better sample storage capability before measurement. The dried method led to the loss of ethanol and methanol and other volatile metabolites. However, the increased dynamic range of the NMR spectrometer (the use of higher receiver gain values) allowed for the enhancement of the remaining non-volatile ^1^H-NMR signals, thus providing a better definition of the ^1^H resonances deriving from the lower concentration molecules present in wines. Despite the achieved improvement in the quality of the ^1^H-NMR spectra, the drying process also results in loss of volatile and semi-volatile compounds, altering thus the chemical composition of the original matrix. Moreover, re-solubilization of the dried samples can be a significant problem, especially if the new solvent has different chemical properties with respect to the original matrix. In this case the metabolite pool would not be longer representative [34]. The observed lower distribution range in the box plot graph could partially be ascribed to this phenomenon.

Numerous primary and secondary metabolites were identified in the ^1^H-NMR spectra of wine samples including amino acids, organic acid, carbohydrates, phenolic compounds, as well as higher alcohols. Amongst these, organic acids are important molecules that contribute to the composition, stability, and organoleptic qualities of wines [35]. The origin of the organic acid pool in wines can be ascribed to two different sources, namely the grapes and the fermentation process. Several organic acids derived from the wine fermentation by yeasts and bacteria were identified in the wine samples including succinic acid, acetic acid, and lactic acid, the final product of the malolactic fermentation. Grapes ripened in a cool climate will normally contain relatively high levels of malic acid, and, therefore, high levels of lactic acid will be reached after the malolactic fermentation. However, 2016 was an unusually warm year with the 5th most sunny autumn ever recorded, thus levels of lactic acid were low to moderate (Mean = 2.14 g/L, Table 2). Tartaric acid, along with malic acid and citric acid, are grape-derived acids that account for the majority of the acidity in wine grapes [35]. The metabolite 2,3-butanediol, a by-product of the alcoholic fermentation and important source of aroma, was found in all the wines samples [36]. The analyzed cool-climate wines were also characterised by the presence of a variety of free amino acids and sugars , which are important nutrients for yeasts during the fermentation process [37]. Amongst the free amino acids, the most predominant were proline and arginine, while phenylalanine and alanine were less abundant in the wine samples. Finally, several higher alcohols were identified in the ^1^H-NMR spectra of wines, including 2-phenylethanol. Higher alcohols represent an important category of aroma compounds in wines that are produced by yeasts using sugars and amino acids as substrates [38].

In order to account for the contribution of minor components to the variability within the analyzed wines, PCA was performed on three different spectral regions of the NMR spectra, namely the aliphatic, carbohydrates and aromatic regions. NMR data normalization and scaling have been omitted in order to be able to compare the real wine chemistry amongst different wines and not only the relative wine chemistry amongst different wines. Data normalization, which has been the default in many wine NMR studies, is required when less standardized sample preparation and data acquisition are performed. In those cases, the proton density (excluding water protons) is assumed to be the same in diluted and full-bodied wines. Therefore a diluted wine will become identical to a powerful concentrated wine, if the relative wine chemistry is the same. The results of the applied PCA showed that the aliphatic and aromatic regions were found to contain most of the systematic variance in the ^1^H-NMR datasets. Well-defined sample groupings according to the wine types were achieved by modelling the abovementioned regions in which organic acids, amino acids, and phenolic compounds are the major contributors to the molecular fingerprint of the different cool-climate wines. ‘Baco Noir’ wines were found to be the richest in lactic acid followed by the two cultivars ‘Prècose Noir’ and ‘Triomphe d’Alsace’. All tree cultivars are old French hybrids derived from crossing *Vitis vinifera* and *Vitis riparia*. The second grape species gives very high acid levels, especially malic acid that, after malolactic fermentation, will result in high levels of lactic acid. The close relationship between these cultivars could also be observed in the PCA scores plots of the aliphatic regions where ‘Précose’ and all the ‘Triomphe D’Alsace’ samples clustered together. Close genetic background may also be observed in the PCA of the aromatic region where ‘Golubok’ cluster together with all the ‘Rondo’ samples. Both cultivars are hybrids of ‘Précose de Malingre’ with a *Vitis amurensis*.

PCA applied to the carbohydrates region did not reveal any well-defined clusters according to different wine varieties, suggesting that the sugar content and type are rather similar in all the analyzed cool-climate wines. The fermentation process also very strongly modifies the original sugar composition of the grapes. Glycerol, one of the major metabolites responsible for wine viscosity [39], and an osmoprotectant produced by yeast during ethanol fermentation, accounted for the majority of the systematic variation in the carbohydrates region. ‘Vinoslivy’ is a Ukrainian hybrid that is characterised by early grape ripening and contains high sugar levels, which, after fermentation, resulted in the highest levels of ethanol and glycerol. Amongst the cultivars with the lowest alcohol level, ‘Bolero’ showed a rather high variability between the repetitions (Table 2) indicating variable fruit quality. Analysis of the sugar content of the juice (free run after crushing) showed 8 g/L difference between samples for ‘Cabernet Cortis’ and up to 9 g/L in ‘Bolero’ (data not shown). The sugar levels obtained in the grapes highly depend on the cultivar, but also on the growing technique. ‘Bolero’ is a cultivar with very big clusters (over 400 g/cluster) and a tendency to “over crop” if not carefully thinned. This may cause variable quality from plant to plant. Some cultivars may also have a tendency to develop bunch stem necrosis, which causes variable quality amongst clusters on the same plant. ‘Cabernet Cortis’ and ‘Rondo’ are prone to this problem, and it may have caused variable ethanol levels amongst replicates. During fermentation, deviations may also develop due to the activity by other microorganisms than the inoculated strain. This, however, appears only to play a minor role, as the estimation of the potential alcohol based on the glucose + fructose content of the juice sampled (Pot. % vol. = g sugar/17) showed an average deviation of only 0.1% vol. if compared to the final wine shown in Table 2. 

The consistency between the ^1^H-NMR and the infrared measurements obtained on the WineScan was assessed by comparison of the ethanol (wet wines), lactic acid and glycerol concentrations. The results showed a high consistency between the two approaches, especially in the case of the wet wines samples. The lower correlation of the glycerol could be ascribed to additional measurement errors due to signal overlapping. Naturally, these prediction models are associated with a prediction error that will depend on the reference method used for infrared calibration. However, in the present study, it is assumed that the WineScan instrument is accurate to within the range of the error of the underlying reference analysis itself, and that the error contributed by the prediction is thus on par or lower in the context of comparison to the NMR data. This is supported by the prediction accuracy of the WineScan for the ethanol prediction of 0.09 vol. % (data not shown).

## 4. Materials and Methods

### 4.1. Experimental Design

**Figure 6:**
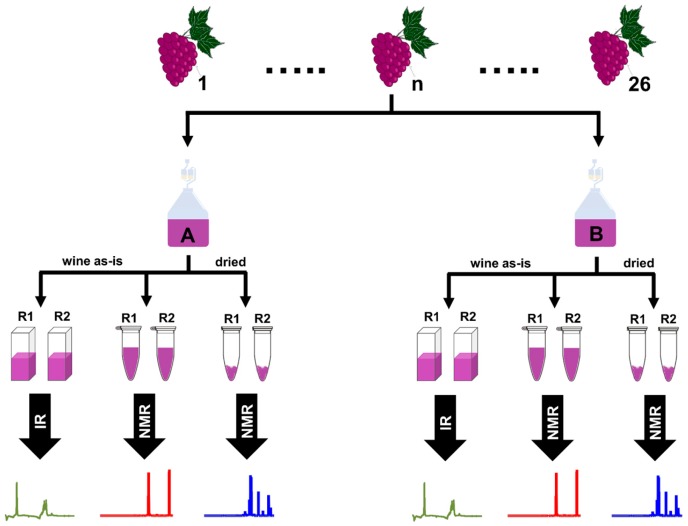
Experimental design for wine analysis. A total of 26 experimental wines were made by using 22 grape varieties. For each experimental wine, two fermentation replicates were prepared (A and B), giving a total of 52 experimental wines. Wine samples were analyzed by FT-IR (wine as-is) and ^1^H-NMR spectroscopy (wine as-is and dried wines). Samples for NMR and IR analysis were prepared in duplicates (R1 and R2).

A total of 22 varieties of cool-climate wine grapes were harvested in vintage 2016 from the field vineyard at the Pometum (Taastrup, Denmark), the fruit and berry Genebank, and experimental orchard of the University of Copenhagen. For each grape variety, the harvested grapes were split in two batches of 10 to 20 kg and processed independently (destemmed and crushed), resulting in two biological wine fermentation replicates (A and B). All wines were inoculated with a commercial *Saccharomyces cerevisiae*, *bayanus* (Lalvin DV10^TM^, Lallemand, Denmark), followed by a malolactic fermentation using Viniflora CH35 (Chr. Hansen, Denmark). 2 g/10 L of the yeast nutrient product Go-Ferm (Lallemand, Denmark) were added during rehydration of the yeast. After 12 days of skin contact with a daily cap punch down, the wines were pressed with a small 20 L hydropress (Speidel, Germany) and transferred to 5 L glass demijohns. The final wines were racked and stabilized with SO_2_ addition. Temperature was kept at 25 °C during fermentations and reduced to 16–18 °C afterwards. Approximately 6 months after harvest, WineScan measurements (WineScan FT 120, FOSS A/S, Hillerød, Denmark) were performed and the wines bottled into 375 mL bottles with screwcap. Samples were stored at 4 °C before NMR measurements. The experimental wine samples were prepared in duplicates for the ^1^H-NMR analysis. In Figure 6 a schematic representation of the experimental design is shown. See Table 2 for the complete list of the grape varieties included in the study.

### 4.2. Chemicals

Deuterium oxide (D_2_O, 99.9%), sodium phosphate monobasic (NaH_2_PO_4_), and sodium 3-trimethylsilyl-propionate-2,2,3,3-d_4_ (TSP) were purchased from Sigma-Aldrich (Darmstadt, Germany).

### 4.3. ^1^H-NMR Spectroscopy

#### 4.3.1. Sample Preparation

*Wet wine.* In order to minimize the chemical shift fluctuation in the ^1^H-NMR spectra due to different pH values in different wine samples, 700 µL of wine sample were transferred to a 1 mL Eppendorf tube containing 300 µL of 1M KH_2_PO_4_ buffer in D_2_O (4:1 *v*/*v*, pH = 3.50 ± 0.02). The obtained mixture was vortexed for 1 minute at maximum speed to ensure sample homogeneity. Subsequently, 600 µL of the sample were transferred to a 5 mm NMR glass tube.

*Dried wine.* For each wine, 1 mL of sample was placed in an Eppendorf tube and dried overnight in a Scanvac centrifugal vacuum concentrator (Labogene, Lynge, Denmark) operating at 25 °C and 1200 rpm. The dried sample was re-dissolved in 600 µL of 1M KH_2_PO_4_ buffer and vortexed for 5 min at maximum speed. The homogenized sample was transferred to a 5 mm NMR glass tube.

#### 4.3.2. ^1^H-NMR Measurements

^1^H-NMR spectra of the wine samples were recorded on a Bruker Avance III 600 operating at a proton Larmor’s frequency of 600.13 MHz and equipped with a 5-mm broadband inverse (BBI) probe. Data acquisition and processing were carried out with the TOPSPIN software (version 3.5). A 5-min waiting period was applied for temperature equilibration prior to ^1^H-NMR measurements. ^1^H-NMR spectra were acquired at 298 K using the standard pulse sequence for presaturation of the water signal (*zgcppr* pulse program), a sweep width of 12,626 Hz, a 90° pulse, and an acquisition time of 3 s. Data were collected into 64 k data points after 256 scans. The relaxation delay (d1) was set to 4 s. The receiver gain (RG) was fixed for all the experiments to an adequate value estimated through several tests for the wet and dried wine samples. The spectra were acquired in automation using the sample jet system (Bruker BioSpin, Ettlingen, Germany). Spectral chemical shift referencing on the TSP CH_3_ signal at 0.00 ppm was performed on all spectra. Metabolites assignments was performed by comparison with the yeast metabolome database (http://www.ymdb.ca/) and with literature data.

### 4.4. Multivariate Analysis

#### 4.4.1. Preprocessing of the ^1^H-NMR Data

**Figure 7:**
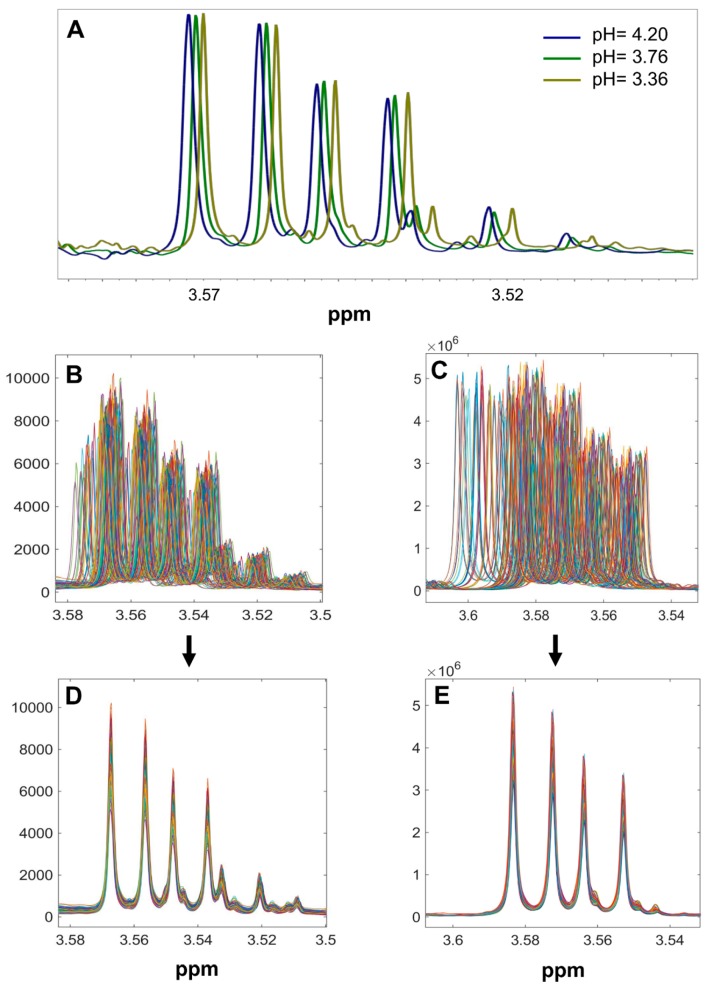
Expansion of the glycerol signal in the ^1^H-NMR spectra of the wine samples at 3 different pH values (**A**); expansion of the glycerol signal in wet wines (**B**–**D**) and dried wines (**C**–**E**) before and after alignment, respectively.

The ^1^H-NMR spectra were imported into the Matlab software (version 2017b, Mathworks Inc., Natick, MA, USA) and two data matrices sized 124 × 65,536 (samples × variables) were built. Spectra were aligned using the *i*coshift algorithm, which is based on correlation optimized shifting of spectral intervals and aligns all spectra simultaneously [40]. Figure 7 shows the ^1^H-NMR spectra of wet and dried wine samples before and after alignment.

The regions containing the residual water signal as well as the noisy “baseline” regions at 9.30–15 ppm and −5–0.8 were excluded from the analysis, making the final data matrices sized 124 samples × 26,001 variables. The ethanol signals were also excluded from the wet wine matrix, yielding a final matrix sized 124 samples × 24,998 variables in the case of wet wines.

The datasets were imported into the PLS_toolbox (version 7.5.1, Eigenvector Research, Mahson, WA, USA) running in MATLAB (version 2017b, Mathworks Inc., Natick, MA, USA). Due to the use of highly standardized protocols for sample preparation, no preprocessing method other than alignment and mean centering was performed prior to multivariate analysis. This is inspired by the extreme standardization efforts made in the metabolomics area for measurements of blood in order to be able to predict lipoprotein distributions across cohorts [41]. 

#### 4.4.2. Principal Component Analysis (PCA)

PCA is an unsupervised method for reducing the dimensionality of a data set consisting of a large number of interrelated variables while retaining the variation present in the data set as much as possible [42]. Reduction is achieved by projecting the variance in the dataset onto orthogonal latent structures called principal components (PCs). The PCs represent the systematic variation of the dataset while noise is isolated as a residual of the model. Samples are assigned scores that correspond to the variation along the PCs. Similar samples will have similar scores and cluster together. The loadings identify the relation between the original variables and define the direction of the PCs in the model space. The results of this exploratory tool is traditionally displayed as scores and loadings plots.

In this study, PCA was applied to explore the systematic variation present amongst the wine samples to discover sample groupings and to evaluate the reproducibility of the sampling protocols. The emerging group structures in the scores plots were evaluated by investigating the chemical information in the loadings plots. The analysis was carried out separately on the aliphatic (0.80–3.00 ppm), carbohydrates (3.01–5.50 ppm), and aromatic (5.51–9.30 ppm) regions of the ^1^H-NMR spectra of two types of datasets separately.

#### 4.4.3. Quality Control of the ^1^H-NMR Data

The consistency of the results of the ^1^H-NMR analysis, based on the two different protocols for samples preparation, was assessed by comparison to Fourier transform infrared (FT-IR) spectroscopy data obtained from the WineScan instrument (FOSS, Hillerød, Denmark) designed for the routine and high throughput chemical analysis of wines. The WineScan estimates the absolute concentration of several organic molecules in wine samples through regression models, which are built through multivariate calibration of the FT-IR instrument against traditional chemical analysis of hundreds of compounds. The regression models are commercially available from the instrument vendor who has developed and is maintaining them. The standard grape wine calibrations developed by FOSS were used in the present study. Increased absolute accuracy may be obtained by slope-intercept adaptation of the models based on the local wines in Denmark. The WineScan is also sensitive to the CO_2_ levels in the wine, which in young wines, like in the present case, can be relatively high. To minimize the effect, CO_2_ was reduced in the samples by repeated shaking (3×) of a half full sample vial prior to analysis.

Lactic acid, glycerol, and ethanol were chosen as target signals for the quality control of the ^1^H-NMR data. These signals were integrated in the ^1^H-MNR spectra of the wet samples. Lactic acid and glycerol were used for the quality control of the dried wine dataset. The ^1^H-NMR data were compared to the data from the reference FT-IR method.

In order to assess the metabolites variation across different samples, the relative amounts of lactic acid, succinic acid, choline, glycerol, tartaric acid, and gallic acid were calculated by integrating the corresponding base-line resolved signals in the ^1^H-NMR spectra of the wet and dried wines. The selected metabolites were normalized to the average value of lactic acid, assuming a complete lactate recovery in both methods [43].

## 5. Conclusions

In this work, a total of 26 cool-climate experimental wines made from 22 different grape varieties were evaluated and measured using ^1^H-NMR spectroscopy. Two different sample preparation methods for the analysis of wine samples by NMR spectroscopy were compared with the aim to find an efficient, sensitive, and reproducible protocol. The wine as-is (wet) approach, in which the sample preparation involves only addition of a 1M KH_2_PO_4_ buffer solution to wine, was found to be a faster method for the NMR analysis of wine samples. Moreover, the buffer addition limited considerably the signal shifts in the ^1^H-NMR spectra due to different pH values in the wine samples and only minor alignment correction was required prior to multivariate data analysis. The obtained NMR data provided a comprehensive overview on the molecules characterizing the Danish cool-climate wines. Nevertheless, the presence of the ethanol spin systems in the wet wine samples hampered partially the analysis of the corresponding NMR spectral region and made deletion necessary prior multivariate analysis. To sum up, almost an equal number of molecules can be detected from both, the “wet wine” and the “dried wine” protocols. Both methods proved to be very consistent and reproducible though differences in the recovery of some metabolites between the two methods remained. It was found that the drying step results in the complete removal of many of the volatile compounds including ethanol, methanol, and other unidentified low concentration compounds. Thus, considering the number of identified metabolites and the simplicity of the protocols, the wet wine sampling protocol is generally to be preferred. The present study illustrates how standardized sample handling in the laboratory with advantage can replace assumptions of the samples introduced by post-analysis modification of the data.

## Figures and Tables

**Table 1:** List of the metabolites identified in the ^1^H-NMR spectra of wet and dried wines. The chemical shifts and multiplicity of the assignments are reported.

Peak Numbers	Metabolite	Wet Wine	Dried Wine
1	Valine	1.04 (d)	1.01 (d), 1.05 (d)
2	2,3-butanediol	1.14 (d)	1.15 (d)
3	Isopropanol	1.13 (d)	1.14 (d)
4	Ethanol	1.17 (t), 1.65 (m)	/
5	Lactic Acid	1.39 (d), 4.29 (m)	1.41 (d), 4.34 (m)
6	Alanine	1.48 (d)	1.52 (d)
7	Arginine	1.65 (m), 1.73 (m)	1.66 (m), 1.74 (m)
8	Proline	2.00 (m)	2.02 (m)
9	Acetic Acid	2.08 (s)	2.12 (s)
10	Methionine	2.08 (m)	2.12 (m)
11	γ-aminobutyric acid	2.48 (t)	2.55 (t)
12	Succinic Acid	2.64 (s)	2.70 (s)
13	Choline	3.19 (s)	3.21 (s)
14	Myoinositol	3.29 (t)	3.32 (t)
15	Methanol	3.35 (s)	/
16	Fructose	3.50 (m)	3.50 (m)
17	Glycerol	3.55 (m), 3.78 (m)	3.57 (m), 3.68 (m), 3.81 (m)
18	Tartaric Acid	4.52 (s)	4.58 (s)
19	β-glucose	4.60 (d)	4.61 (d)
20	α-glucose	5.25 (d)	5.26 (d)
21	Arabinose	5.30 (d)	5.30 (d)
22	cis-caftaric acid	5.34 (s)	5.34 (s)
23	Tyrosine	6.88 (d), 7.18 (d)	6.92 (d), 7.20 (d)
24	Gallic Acid	7.14 (s)	7.16 (s)
25	2-pheniylethanol	7.35 (m)	7.37 (m)
26	Phenylalanine	7.40 (m)	7.41 (m)
27	Trigonelline	8.06 (m), 8.84 (m), 9.13 (s)	8.07 (m), 8.83 (m), 9.14 (s)

s: singlet; d: doublet; m: multiplet.

**Table 2:** List of the grape varieties used for making the wine samples. A and B represent different fermentation replicates. A brief description of the grape varieties, pH values, and the absolute concentrations of glycerol, lactic acid, and ethanol, as measured by the WineScan instrument in the wine samples, are reported. Absolute concentrations are expressed as g/L or % volume (ethanol). (L.): Grapes form plants treated with LalVigne Mature at veraison; (Y.): Grapes from young plants.

Grape Variety	Description	Wine Samples	pH	Glycerol (g/L)	Lactic Acid (g/L)	Ethanol (% vol.)
Acolon	‘Acolon’ is a German wine grape variety created in 1971 by crossing ‘Lemberger’ (‘Blau Fränkisch’) and ‘Dornfelder’. Medium to large clusters and berries. It ripens early and produces a very colour-intensive wine with mild tannins.	A	3.56	7.58	2.14	11.58
		B	3.55	7.33	2.20	11.43
Baco Noir	‘Baco Noir’ is a hybrid grape variety produced in France in 1902 by crossing a French variety of *Vitis vinifera* named ‘Folle blanche’ and the native American *Vitis riparia*. Medium clusters with small berries. Wines made from ‘Baco Noir’ are known to be rustic, wild, and great for staining teeth because of their heavy pigment.	A	3.35	5.22	4.04	9.53
		B	3.39	5.36	3.98	9.53
Bolero	‘Bolero’ is an interspecific wine grape variety created in Germany in 1982. It is a cross between (‘Rotberger’ × ‘Reichensteiner’) and ’Chancellor’ grapes. It has *Vitis rupestris* in its pedigree. Large clusters and berries. It ripens quite early and produces a ruby red wine, harmonious on the palate with medium tannins.	A	3.64	5.15	1.88	9.14
		B	3.70	5.69	2.01	10.04
		A (L.)	3.56	5.23	1.82	9.02
		B (L.)	3.42	4.65	1.63	8.33
Cabernet Cantor	‘Cabernet Cantor’ is an interspecific red grape created in 1989 by crossing the grape varieties ‘Chancellor’, ‘Merzling’, ‘Zarya Severa’, and ‘Muscat Ottonel’. It gives a wine with dark berry and black pepper flavour, which is soft but rich in extract and phenolic compounds.	A	3.89	7.40	1.21	11.00
		B	3.88	7.50	1.22	10.91
Cabernet Cortis	‘Cabernet Cortis’ is a dark-skinned interspecific grape variety. It was bred in Germany in 1982 by crossing ‘Cabernet Sauvignon’ and ‘Solaris’. Medium-sized clusters and small to medium berries. It ripens relatively early and produces colourful and tannic wines with vegetal characters, which are very herby on the palate, rich in extract, and contains phenolic compounds.	A	3.78	7.27	2.34	11.81
		B	3.81	7.57	2.22	12.38
Cabaret Noir	‘Cabaret Noir’ is an interspecific grape variety created in Switzerland by the breeder V. Blattner in 1991 by crossing ‘Cabernet Sauvignon’ with an interspecific resistant cultivar. Clusters and berries are small. Produces a wine with cabernet character.	A	4.00	7.71	1.81	12.26
		B	4.07	8.03	1.84	12.65
Don Muscat	‘Don Muscat’ is a Russian cultivar. The genetic origin is unknown to us, but we believe it to be interspecific. It produces a light red wine, very rich in flowery, and fruity muscat flavours.	A	3.93	5.62	2.78	9.22
		B	3.99	5.95	2.88	9.41
Eszter	‘Eszter’ is an interspecific cultivar created in Hungary in 1969 by crossing ‘Eger 2’ and ‘Magaracsi’. It has *Vitis berlandieri* in its pedigree. It produces a light fruity aromatic wine.	A	3.79	7.81	1.60	11.24
		B	3.80	7.92	1.60	11.34
Frühburgunder	‘Frühburgunder’, known also as ‘Pinot Noir Précoce’, is the early ripening version of ‘Pinot Noir’. Clusters are small and tight with small berries. It is an old cultivar mutation likely to originate in France. It gives a light coloured soft and velvet Pinot style wine characterised by a fruity spicy aroma.	A	4.04	8.29	1.96	12.66
		B	4.12	8.25	2.09	12.57
Golubok	‘Golubok’ is a cultivar originating from Ukraine. The genetic background is unknown to us. Clusters and berries are medium sized. In this grape, the colour pigment is not only located in the skin, but a significant amount of pigment is also present in the pulp. This results in a deep, rich, dark, almost black colour and a unique flavour.	A	4.07	8.40	2.32	10.57
		B	4.04	8.51	2.26	10.73
Lemberger	‘Lemberger’ (or ‘Blau Fränkisch’) is an old cultivar assumed to origin in Austria or Franconia in Germany. ‘Heunisch’ is proven to be one of the parents. The tight clusters and berries are medium sized. It produces a well coloured fruity wine with dark berry flavours with a bit spicy character. The wines are well balanced with a good soft tannin structure.	A	3.47	7.83	2.00	11.52
		B	3.45	8.04	2.01	11.74
Leon Millot	‘Léon Millot’ is an old French hybrid created in 1911 in Alsace by crossing the hybrid grape ‘Millardet et Grasset’ (*Vitis riparia* × *Vitis rupestris*) with ‘Goldriesling’, which is a *Vitis vinifera* variety. The relatively early ripening makes it particularly suited for cultivation in cool climates. Clusters and berries are small. Common aromatic and flavour profiles for ‘Leon Millot’ include earthy, barnyard, woody notes, purple fruits, and chocolate.	A	4.15	7.87	1.95	11.59
		B	4.20	7.81	2.06	11.51
Monarch	‘Monarch’ is an interspecific grape variety breed in Germany in 1988. It is a crossing of ‘Solaris’ and ‘Dornfelder’. From Solaris it has several Vitis species in its pedigree. It produces a light fruity wine with red and dark berry flavours.	A	3.42	6.04	1.74	9.53
		B	3.45	6.03	1.75	9.54
Nero	‘Nero’ is an interspecific cultivar originating in Hungary. It was created in 1965 by crossing (‘Medoc Noir’ x ‘Perle von Csaba’) and (‘S.C 12375’ × ‘Gárdonyi’). Clusters and berries are medium to large and may be used as table grape. It produces a light aromatic wine with red berry flavours with a little muscat character.	A	3.78	7.63	2.10	11.91
		B	3.76	7.68	2.09	11.87
New York Muscat	‘New York Muscat’ is a cultivar made in US at Cornell University by crossing ‘Muscat Hamburg’ and ‘Ontario’. Clusters and berries are medium in size and may be enjoyed as table grape. It produces a deep red very aromatic wine with floral muscat flavours.	A	3.86	5.94	2.07	10.37
		B	3.90	6.01	2.07	10.25
Précose Noir	‘Précose Noir’ is an old French hybrid from the same breeding as ‘Triomphe d’Alsace. Clusters and berries are small to medium. Produces a medium dark red light bodied wine with dark berries and spicy aromas.	A	3.90	7.21	2.90	11.42
		B	3.88	7.20	2.89	11.39
Reberger	‘Reberger’ is an interspecific grape variety that was breed in Germany in 1986 by crossing the ‘Regent’ and ’Lemberger’ (‘Blau Fränkisch’). The loose clusters and berries are medium sized. It produces a light fruity wine with a tannic/phenolic character.	A	3.83	7.44	2.20	12.03
		B	3.70	7.17	2.11	11.73
Regent	‘Regent’ is an interspecific grape variety created in Germany in 1967 by crossing ‘Diana’ (‘Silvaner’ × ‘Müller-Thurgau’) with the hybrid ‘Chambourcin’. Clusters are relatively loose medium in size with small to medium berries. It gives colourful and tannic wines and shows aromas of cherries or black currants with peppery notes.	A	3.89	7.39	2.19	12.22
		B	3.88	7.45	2.15	12.23
Rondo	‘Rondo’ is an interspecific variety created in 1964 in then-Czechoslovakia by crossing ‘Zarya Severa’ × ‘St. Laurent’. It is characterised by a very early ripening which make it particularly suitable for cultivation in cool climates. Clusters and berries are medium sized. ‘Rondo’ produces a very dark red wine with dark berry and woody aromas.	A	3.81	8.18	1.52	11.67
		B	3.73	8.15	1.32	11.75
		A (L.)	3.75	8.30	1.75	11.30
		B (L.)	3.83	8.30	1.83	11.41
		A (Y.)	3.73	8.03	1.78	11.87
		B (Y.)	3.77	8.08	1.85	11.84
Titan	‘Titan’ is a cultivar developed at the research station in Eger in Hungary. The genetic origins is unknown to us, but believe it to be interspecific. The berries are characterised by coloured flesh resulting in a very dark almost black wine. Clusters and berries are medium in size. The wine has good dark berry flavours and rich good phenolic structure.	A	3.92	8.18	2.13	10.85
		B	3.93	8.13	2.09	10.85
Triomphe d’Alsace	‘Triomphe d’Alsace’ is an interspecific grape variety that was produced in France in 1911 by crossing the American grape species *Vitis riparia* with *Vitis rupestris*. The resultant hybrid was then crossed with ‘Goldriesling’ (*Vitis vinifera)*. Clusters and berries are small. It produces a medium dark red light bodied wine with dark berries and spicy aromas. Quite similar to ‘Prècose Noir’.	A	3.70	6.33	3.16	10.89
		B	3.76	6.83	3.11	11.43
		A (L.)	3.81	6.76	2.95	11.33
		B (L.)	3.63	6.34	2.95	11.00
Vinoslivy	’Vinoslivy’ is an interspecific cultivar breed in Ukraine in 1958 by crossing ‘Getsh’ with (‘Muscat Hamburg’ × *Vitis amurensis*). It is very early ripening and accumulates relatively high sugar levels. The clusters and berries are small to medium. It produces a light red wine with an aroma rich in pyrazines.	A	3.64	8.54	1.36	13.09
		B	3.61	8.42	1.37	12.92
Average			3.78	7.23	2.14	11.16
Max			4.20	8.54	4.04	13.09
Min			3.35	4.65	1.21	8.33
